# Dissecting AlphaFold2’s capabilities with limited sequence information

**DOI:** 10.1093/bioadv/vbae187

**Published:** 2024-11-25

**Authors:** Jannik Adrian Gut, Thomas Lemmin

**Affiliations:** Institute of Biochemistry and Molecular Medicine, University of Bern, Bern 3012, Switzerland; Graduate School for Cellular and Biomedical Sciences (GCB), University of Bern, Bern 3012, Switzerland; Institute of Biochemistry and Molecular Medicine, University of Bern, Bern 3012, Switzerland

## Abstract

**Summary:**

Protein structure prediction aims to infer a protein’s three-dimensional (3D) structure from its amino acid sequence. Protein structure is pivotal for elucidating protein functions, interactions, and driving biotechnological innovation. The deep learning model AlphaFold2, has revolutionized this field by leveraging phylogenetic information from multiple sequence alignments (MSAs) to achieve remarkable accuracy in protein structure prediction. However, a key question remains: how well does AlphaFold2 understand protein structures? This study investigates AlphaFold2’s capabilities when relying primarily on high-quality template structures, without the additional information provided by MSAs. By designing experiments that probe local and global structural understanding, we aimed to dissect its dependence on specific features and its ability to handle missing information. Our findings revealed AlphaFold2’s reliance on sterically valid Cβ for correctly interpreting structural templates. Additionally, we observed its remarkable ability to recover 3D structures from certain perturbations and the negligible impact of the previous structure in recycling. Collectively, these results support the hypothesis that AlphaFold2 has learned an accurate biophysical energy function. However, this function seems most effective for local interactions. Our work advances understanding of how deep learning models predict protein structures and provides guidance for researchers aiming to overcome limitations in these models.

**Availability and implementation:**

Data and implementation are available at https://github.com/ibmm-unibe-ch/template-analysis.

## 1 Introduction

Proteins serve as nano-machines within cells, orchestrating a plethora of vital functions essential for life. Their remarkable versatility arises from their unique three-dimensional (3D) structures, dictated by the specific sequence of amino acids they are built from. This fundamental principle, known as Anfinsen’s dogma (Anfinsen 1973), underpins modern biology and fuels research in areas like drug discovery and enzyme engineering. However, progress in elucidating protein structures has been hindered by the labor-intensive nature of *in vitro* experiments required for atomic structure determination, resulting in only approximately 200 000 structures being resolved to date (Burley *et al.* 2017).

To surmount this bottleneck, researchers have increasingly turned to computational methodologies to unravel the intricacies of protein folding. Established in 1994, the Critical Assessment of Techniques for Protein Structure Prediction (CASP) (Defay and Cohen 1995) has played a crucial role in tracking advancements in this field. Recent years have been particularly transformative, fueled by two key factors: the exponential growth of sequential and structural protein data (Burley *et al.* 2017, UniProt-Consortium 2019) and the emergence of powerful machine learning methodologies, particularly deep learning, capable of harnessing this data more effectively. Notably, AlphaFold2 (Jumper *et al.* 2021), a deep neural network unveiled in 2020, has revolutionized the field. This innovative model has achieved remarkable accuracy in predicting protein structures, marking a significant leap forward in our understanding of protein structure and function.

The AlphaFold2 pipeline follows a two-step process for predicting protein structures. First, it searches various protein sequence databases (Suzek *et al.* 2015, Burley *et al.* 2017, Mirdita *et al.* 2017, UniProt-Consortium, 2019, Mitchell *et al.* 2020) using dedicated tools (Finn *et al.* 2011, Lassmann 2019, Steinegger *et al.* 2019) to find similar sequences to the target protein across. This information gets compiled into a multiple sequence alignment (MSA), capturing the evolutionary relationships between the proteins. Simultaneously, AlphaFold2 identifies suitable 3D structures (templates) from closely related proteins to serve as initial structural models. These two sources of information, MSA and templates, are initially processed separately within the AlphaFold2 model. However, their representations are continuously refined through an iterative exchange of information, allowing the model to learn from both sources simultaneously. Finally, the refined representations of the MSA and templates are combined in the structure module of AlphaFold2 to generate the final protein structure and assign a confidence score (pLDDT) for each individual amino acid.

The MSA has been observed to play a more significant role in predicting protein structure quality compared to templates (Zhang *et al.* 2023, Guan *et al.* 2024, Mirabello *et al.* 2024, Monteiro da Silva *et al.* 2024, Wayment-Steele *et al.* 2024). Notably, several pipelines leveraging AlphaFold2 (Del Alamo *et al.* 2022, Wallner, 2023), and three out of the five default models of AlphaFold2, ignore the information from templates altogether.

Intriguingly, contrary to previous pipelines, AF2Rank (Roney and Ovchinnikov 2022) showed that simply providing AlphaFold2 with a protein structure, without any sequence information, can be used to evaluate its plausibility and discriminate between real structures and decoys. The authors hypothesized that the structure module within AlphaFold2 has learned a robust biophysical energy function, and the MSA input might primarily serve to guide the model toward the correct energy minimum.

To further evaluate this hypothesis, we conducted an extensive investigation into the influence of the template input and structure recycling on AlphaFold2’s predictive accuracy. Through a comprehensive series of ablation studies, we assessed the model’s capability to reconstruct protein structures solely based on structure input, without relying on deep MSAs. Specifically, we examined AlphaFold2’s performance in side-chain packing and its resilience in recovering from artificially perturbed proteins. Our experimental code is openly accessible on GitHub (https://github.com/ibmm-unibe-ch/template-analysis) and we have contributed new methods to the OpenFold (Ahdritz *et al.* 2024) project (https://github.com/aqlaboratory/openfold/pull/408).

Our findings offer valuable insights for both AlphaFold2 users and developers who integrate the tool into their workflows. By enhancing the understanding of the model’s limitations and providing guidance on result interpretation, our work aims to empower users to critically assess results and leverage complementary tools when necessary. Additionally, it encourages exploration of existing tools or the development of innovative solutions to address these limitations, ultimately contributing to more accurate and reliable protein structure predictions.

## 2 Materials and methods

### 2.1 Datasets

The data used for the experiments were sourced from CASP13 (Kinch *et al.* 2019, Kryshtafovych *et al.* 2019), CASP14 (Kinch *et al.* 2021, Kryshtafovych *et al.* 2021), and CAMEO targets from September 2024 (Robin *et al.* 2021). CASP13 was included in the analysis to evaluate potential bias and overfitting, given that AlphaFold2 was trained on proteins within the CASP13 dataset. Consequently, we anticipate observing higher scores compared to CASP14, which serves as a more realistic benchmark for assessing the model’s performance on unseen targets. While the CAMEO dataset provides a continuous evaluation of the model’s performance, it may include structures that are homologous to those in AlphaFold2’s training set. These distinct datasets are crucial for accurately gauging the model’s generalizability and effectiveness in predicting structures of novel protein sequences.

### 2.2 AlphaFold2

We primarily employed the LocalColabFold (https://github.com/YoshitakaMo/localcolabfold) (Mirdita *et al.* 2022) implementation of AlphaFold2 in our experiments, as it offers a user-friendly command-line interface and faster inference enabled by the MMseqs webserver (Mirdita *et al.* 2021). Given that AlphaFold2 largely ignores template information when provided with a deep MSA, a minimal MSA comprising only the query sequence (single sequence) was supplied to the model, unless otherwise specified. Furthermore, we developed OF2Rank, a protocol within the OpenFold (Ahdritz *et al.* 2024) framework similar to AF2Rank that was previously used to assess the quality of protein structures (Roney and Ovchinnikov 2022). This method involves replacing all original amino acid information in the template with glycine residues extended with a Cβ. The sequence information is provided either as an all-gap MSA (*Gaps*) or as a single sequence MSA (*Single*). Due to technical constraints, OF2Rank was provided with the original amino acid sequence as an input sequence instead of gaps.

One key idea of AlphaFold2 is the recycling mechanism. This process iteratively refines protein structure predictions by feeding back the MSA embedding, pair embedding, and the structure prediction (*prev_x*) of the previous iteration into the model. To evaluate the impact of a pre-existing template on predictions to this recycling, we modified OpenFold. This modification allows us to introduce a custom template structure as the “previous prediction” during the very first iteration of the recycling process (denoted as “-1”).

### 2.3 Side-chain packing

To assess AlphaFold2’s ability to reconstruct side-chains accurately, we designed four test cases using CASP13, CASP14, and CAMEO data. In each case, all side-chain atoms were removed from the target proteins. In addition, to providing just the backbone as a template, we also positioned the Cβ in three different ways:

(i) *Non-informative C*β: Placed next to the origin for a baseline comparison, (ii) *Heuristic C*β: Predicted using a rule-based approach based on the backbone atoms (Roney and Ovchinnikov 2022), and (iii) *Template C*β: Maintained the original Cβ position from the template.

We extended our assessment to explore AlphaFold2’s ability to refine side-chain predictions using three external side-chain packing strategies: the lowest energy conformation provided by the widely used CHARMM 36 force field (*C36*), FASPR (Huang *et al.* 2020), a structure-based side-chain packing method and AttnPacker, a neural network-based side-chain packing method (McPartlon and Xu 2023).

The modified templates and single sequences were then inputted into LocalColabFold without relaxation and without recycling, ensuring that the backbone remained more similar to the input template. Only the predictions from the first two AlphaFold2 models were considered, as they are the ones where template input is utilized.

### 2.4 Structural perturbation

**Figure 1.**
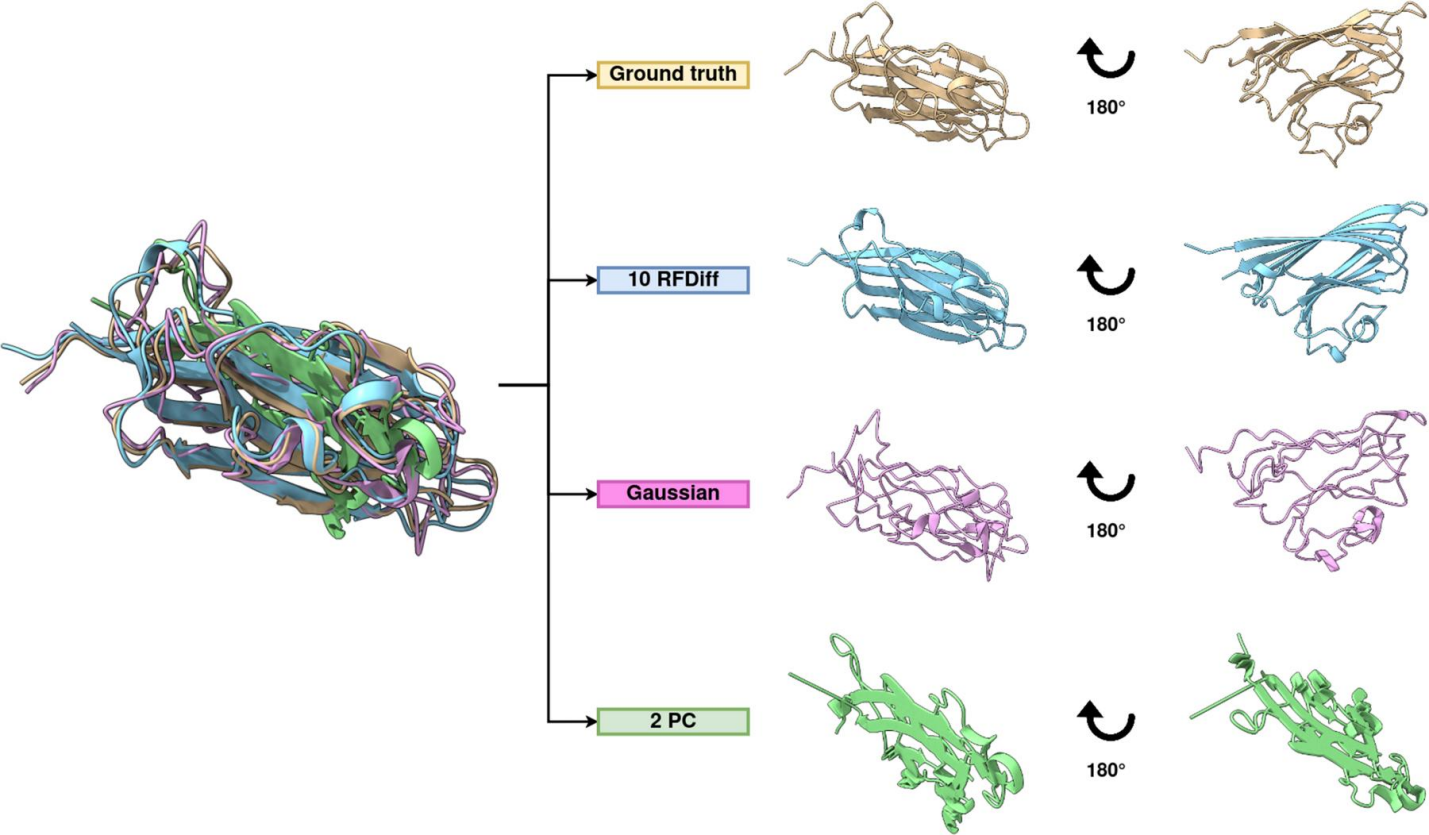
Illustration of structural perturbations. Ground truth protein structure (T1026, PDB id: 6s44) is shown in yellow on top, 10 partial RFdiffusion steps in blue on below, Gaussian noise in pink on below, and 2D projection in green at the bottom. All structures were rendered using ChimeraX (Meng *et al.* 2023).

Three distinct techniques were employed to generate controlled perturbations to the template structures (Fig. 1). The refinement of perturbed structures was conducted using LocalColabFold with either a single sequence or MSA. We also evaluated the performance of this approach as a homology modeling pipeline. Finally, we tested the newly implemented protocol within OpenFold to evaluate a more sequence-agnostic refinement.

#### 2.4.1 Gaussian noise

The simplest perturbation method involved adding Gaussian noise to the template coordinates. We independently sampled values from a standard normal distribution (mean = 0 Å) for each atomic coordinate dimension. A predefined standard deviation of 1 Å determined the magnitude of the introduced noise. These sampled values were then added to the original coordinates, introducing controlled deviations from the initial structure.

#### 2.4.2 Principal component analysis

Principal component analysis (PCA) (Bishop 2007) was employed to transform and project the protein into an orthogonal reference frame that explains the most variance. Separate PCAs were computed for each protein in the CASP13, CASP14, and CAMEO datasets. Subsequently, each structure was projected onto the subspace defined by the first principal component (*1 PC*) or the first two principal components (*2 PC*). This effectively captured the major structural variations within each protein structure.

#### 2.4.3 RFdiffusion

RFdiffusion (Watson *et al.* 2023) leverages diffusion techniques (Sohl-Dickstein *et al.* 2015) to generate new proteins from noise. Instead of executing the complete diffusion process, which generates entirely new backbones conformations, we ran RFdiffusion for a limited number of partial steps (1, 5, and 10 steps) (Torres *et al.* 2024). This strategy produced backbones that remained closer to the starting structure, with the degree of variability increasing with the number of steps. Subsequently, side-chains were reconstructed using FASPR or AttnPacker.

#### 2.4.4 Homology modeling

We utilized MMseqs2 (Mirdita *et al.* 2021) to search for one template for each protein in the CAMEO dataset. Templates were selected from the PDB databank (Burley *et al.* 2017) based on a sequence identity between 30% and 70% and a sequence coverage of at least 80%. These templates along with the corresponding query sequence were then inputted into LocalColabFold.

### 2.5 Evaluation metrics

To assess the accuracy of the predicted protein structures, we employed a variety of metrics. These metrics can be broadly categorized into three classes based on the level of detail they capture.

The first class of accuracy metrics focuses solely on the α-carbon atom from the backbone. This includes the Template Modeling (TM)-score (Zhang and Skolnick 2004, Biasini *et al.* 2013), which quantifies the structural similarity between predicted and reference structures, and the α-RMSD (Biasini *et al.* 2013), measuring the rectified root mean squared deviation between the Cα positions of the predicted and reference structures after optimal superposition using the Kabsch algorithm.

The next class of accuracy metrics encompasses RMSD and the Local Distance Difference Test (lDDT) (Biasini *et al.* 2013, Mariani *et al.* 2013), which evaluates all atom pairs (backbone and side-chain) within a predefined radius excluding those belonging to the same residue. In addition, AlphaFold2 predicts a score called pLDDT, which estimates the lDDT value of its generated structures and helps guide the selection of the best model among multiple predictions. Individual per-residue lDDT scores can be combined to generate a single, global lDDT score. To accommodate potentially invalid protein structures with steric clashes, stereochemical checks were disabled during lDDT calculations. When dealing with structures with difference sequences, the default Needleman-Wunsch (Needleman and Wunsch, 1970) algorithm using BLOSUM62 (Henikoff and Henikoff 1992) was used to align the sequences.

The last class of metrics evaluates the accuracy of side-chain packing with the Mean Absolute Error for the first four dihedral angles of the side-chains (McPartlon and Xu 2023).

The preprocessing and extraction of gapless sequences from PDB structures were performed using Pdb-tools (Rodrigues *et al.* 2018) and PDBFixer (Eastman *et al.* 2017). The RCSB MAXIT(Burley *et al.* 2017) suite was employed to translate from PDB files to CIF format. ProDy (Bakan *et al.* 2011) and Scikit-learn (Pedregosa *et al.* 2011) were utilized for implementing the experiments and analyzing the results.

## 3 Results

To assess AlphaFold2’s (Jumper *et al.* 2021) understanding of protein structure, we designed two complementary tasks. The first task assessed AlphaFold2’s local understanding by testing its ability to rebuild and pack side-chains onto a provided backbone template. This evaluated AlphaFold2’s ability to handle individual amino acid interactions within the protein structure. The second task investigated the global understanding by evaluating AlphaFold2’s effectiveness in recovering the correct protein structure from a perturbed template. Previous studies have indicated that AlphaFold2 largely disregards template input when provided with a deep MSA (Adiyaman *et al.* 2023). Therefore, to isolate its capability in utilizing structural information, we primarily employed a single sequence along with the template as input for AlphaFold2 in most experiments. To assess potential overfitting concerns, we compared AlphaFold2’s performance on easier targets, such as the CAMEO dataset (with potential homologs to its training set) and CASP13 (part of its training data), to its performance on the more challenging targets from CASP14. Since we did not observe any major differences between these two datasets, we will focus on presenting the results from CASP14 in this section, but report data of both datasets in Supplementary Section A and Supplementary Section C for side-chain packing and structural refinement respectively.

### 3.1 Side-chain packing

Side-chain packing is a critical process in protein structure prediction, involving the prediction of the 3D positions of amino acid side-chains relative to the protein backbone. This task is essential for accurate modeling of protein structures and for understanding their biological functions.

Our initial evaluation focused on AlphaFold2’s ability to pack side-chains using only the backbone atoms and different approaches for the placement of Cβs: all close to the origin (*Non-informative C*β*)*, using a heuristic (*Heuristic C*β*)*, or preserving the correct position from the template (*Template C*β*)*. When the template lacked Cβ information (either missing or Non-informative Cβ), the predicted structures suffered notable accuracy loss. The average TM-score dropped to approximately 0.41 ± 0.20 and 0.32 ± 0.20, respectively, indicating a failure to preserve the 3D structure. Providing more informative Cβ positions, either through heuristics or the template, substantially improved the results. The average TM-score remained very high (nearly 0.98 ± 0.03) when using the heuristic Cβ placement, indicating minimal alterations of the backbone structure from the template. It is worth noting that this score is higher than the average TM-score achieved by AlphaFold2 predictions using a full MSA without a template (approximately 0.8 ± 0.18).

These high TM-scores suggest that side-chain packing metrics can be reliably analyzed, since the overall protein fold is mostly maintained. The heuristic Cβ placement achieved a promising average lDDT score of 0.88 ± 0.05, exceeding the baseline method (standard pipeline with full MSA) which had an average lDDT of 0.74 ± 0.15. Next, we assessed AlphaFold2’s potential to enhance the predictions of three different methods for side-chain placement: predefined conformations from the CHARMM36 force field, FASPR (Huang *et al.* 2020), and AttnPacker (McPartlon and Xu 2023). We employed the AutoPSF plug-in from VMD (Humphrey *et al.* 1996) to assign a single, predefined side-chain structure to each residue based on the CHARMM36 force field (*C36*). While fast and simple, it neglects the local environment, leading to clashes and sub-optimal packing. FASPR (Huang *et al.* 2020) utilizes a tree search algorithm to identify energetically favorable placements for predefined rotamers (Shapovalov and Dunbrack 2011). This approach offers better balance between speed and accuracy by using a predefined library of conformations but allowing for optimization based on energy minimization. Finally, AttnPacker is a deep learning-based method that directly predicts side-chain coordinates without relying on predefined rotamers. This approach offers greater flexibility in side-chain placement compared to rotamer-based methods.

**Figure 2.**
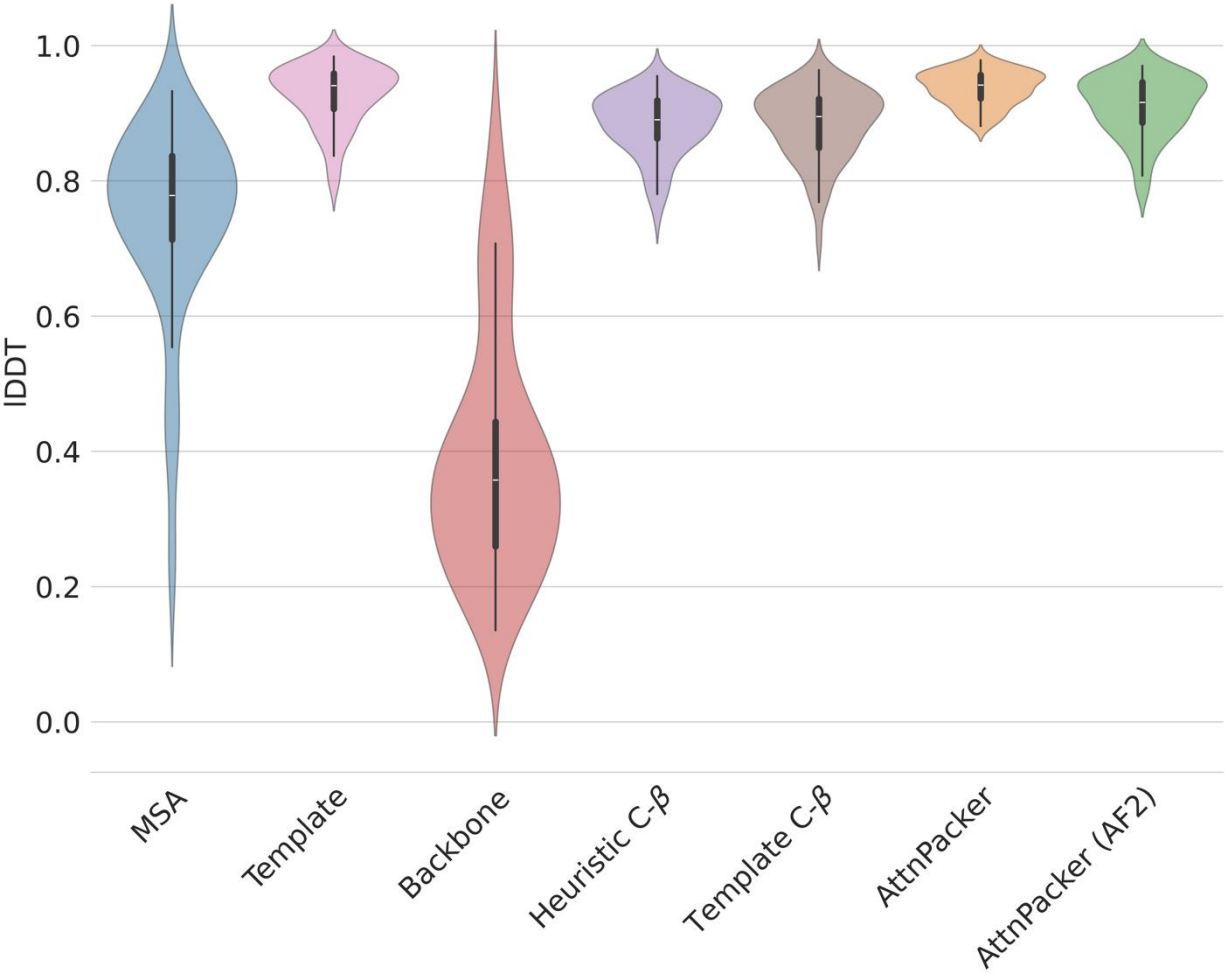
Average lDDT scores for side-chain packing task. Violin plots show the distribution of average lDDT scores for various AlphaFold2 template configurations. *MSA*: full MSA, but no template, *Template*: full template, *Backbone*: the protein backbone, *Heuristic C*β: template with heuristically placed Cβ, *Template with C*β: backbone and Cβ from the ground truth, *AttnPacker* and *AttnPacker (AF2)*: ground truth backbone with side-chains placed by AttnPacker before and after AF2 refinement.

Refining the repacked protein structures through AlphaFold2 resulted in a slight decrease in the average TM-score to 0.98 ± 0.03, indicating again minimal backbone alteration. Interestingly, refining the packing with AlphaFold2 greatly boosted the average lDDT scores when the initial packing was poor (e.g. predefined rotamers from the CHARMM36 force field). However, for methods like FASPR and AttnPacker, which already produced good initial packing, the lDDT scores remained similar or even decreased slightly after AlphaFold2 refinement (Fig. 2).

Analyzing the mean RMSD revealed similar effects of AlphaFold2 refinement on different packing methods. Notably, competitive side-chain packing methods like FASPR and AttnPacker see a marginal change in performance after post-processing with AlphaFold2. Conversely, when using only the default low energy conformation from the CHARMM36 force field, which initially performs poorly (RMSD: 1.77 Å ± 0.15), exhibited a notable improvement in side-chain placement (RMSD: 0.88 Å ± 0.25) after AlphaFold2 refinement. When provided with only the heuristic Cβ, AlphaFold2 is able to predict the side-chain position with a similar precision as FASPR, RMSD 0.91 Å ± 0.25 and 0.90 Å ± 0.27 respectively. While providing the correct Cβ information leads to a marginal improvement (RMSD: 0.86 Å ± 0.27) over the heuristic approach.

Furthermore, we noticed a slight decrease in confidence and precision as residues become more exposed at the protein surface (Supplementary Section B). This decrease is consistent across all template-informed protocols but is notably steeper for the standard AlphaFold2 protocol relying solely on MSAs. However, we did not find any systematic differences related to secondary structure types or the local structural complexity and prediction challenges, as estimated by the pLDDT score from the full MSA-based prediction (Supplementary Section E).

### 3.2 Structural refinement

Next, we assessed AlphaFold2’s ability to refine perturbed templates. Three perturbation protocols were implemented and tested: introducing random Gaussian noise to atom coordinates (*Gaussian*), projecting the entire structure onto a 1D or 2D space defined by principal components (*1 PC* and *2 PC*, respectively), and partially denoising the structure using RFdiffusion (*RFDiff*). The full table with scores can be found in Supplementary Section C.

Despite struggling to fully recover the original structure for several perturbations, AlphaFold2 generated sterically valid structures in most cases. This is evident in the necessity to disable stereochemical checks from OpenStructure (Biasini *et al.* 2013) when calculating lDDT for templates perturbed with the Gaussian noise and projected in to the PCA eigenspace. These checks ensure proper atomic interactions, and the initial violation by a majority of residues suggests greater structural distortion. However, the fact that these checks would not be needed for post-processed structures implies that AlphaFold2, while not achieving complete fold recovery, produces structures with proper atomic interactions.

**Figure 3.**
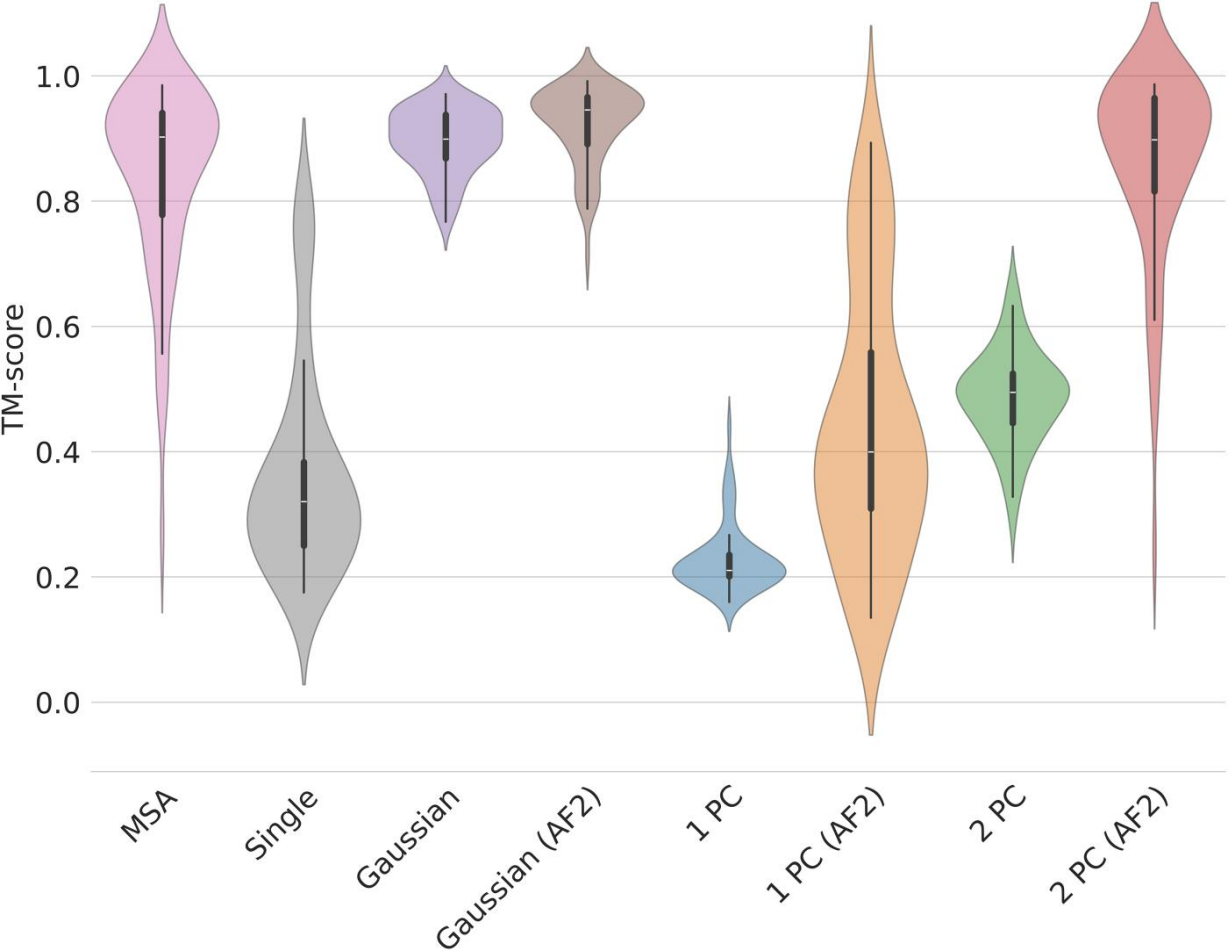
Violin plot of selected perturbation strategies. (*AF2*) is used to identify the post-processing by AlphaFold2, *MSA* uses the standard AlphaFold2 pipeline with a full MSA, while *Single* uses only the single sequence, *Gaussian* adds Gaussian noise to the coordinates, *1 PC* projects proteins to the first principal component, *2 PC* projects targets to the first two principal components.

AlphaFold2 demonstrated good recovery capabilities for templates perturbed with Gaussian noise (Fig. 3). The average TM-score increased from 0.90 ± 0.05 to 0.92 ± 0.06, and the lDDT score improved from 0.66 ± 0.00 to 0.82 ± 0.07. Notably, AlphaFold2 excelled at recovering structures projected onto the two-dimensional PCA space. Here, refinement notably boosted accuracy, with the average TM-score rising from a low 0.49 ± 0.08 to 0.86 ± 0.15 and the mean lDDT increasing from 0.53 ± 0.09 to 0.79 ± 0.12. While refinement also improved results for one-dimensional projections, the final refined scores (average TM-score: 0.44 ± 0.21 and mean lDDT: 0.39 ± 0.20) remained relatively low.

**Figure 4.**
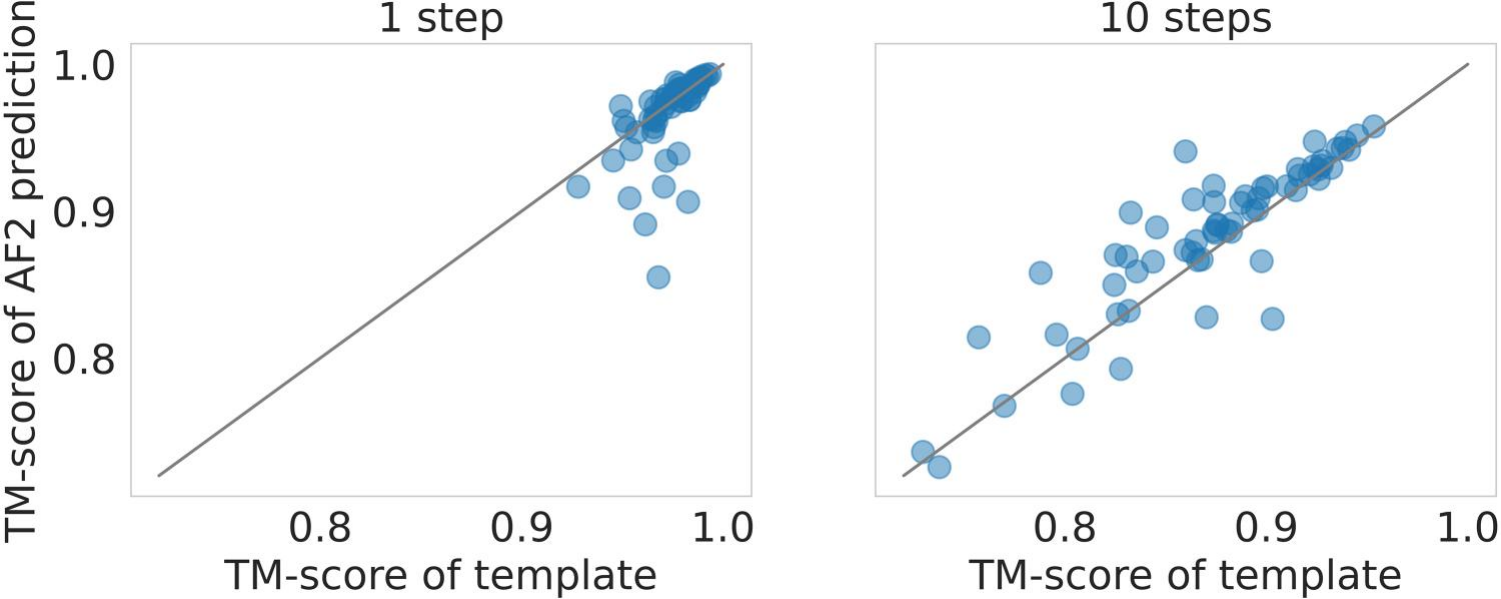
Change in TM-score after AlphaFold2 refinement of RFdiffusion-perturbed templates. Each point represents a single target from the CASP14 dataset. The TM-score of the RFdiffusion-perturbed structure before refinement is displayed on the *x*-axis, with separate plots for 1 and 10 partial steps on the left and right, respectively. The *y*-axis shows the TM-score after refinement using AlphaFold2. The side-chains were packed using FASPR.

For the RFdiffusion-based perturbation, we observed a gradual decrease in both TM-score and lDDT, as the number of partial diffusion steps increased. The average TM-score dropped from 0.97 ± 0.01 with one diffusion step to 0.87 ± 0.05 with 10 steps (Fig. 4), while the average lDDT fell from 0.80 ± 0.03 to 0.67 ± 0.05. These scores suggest that despite the perturbation, the protein remained within the same general fold. This implies that the starting point for refinement by AlphaFold2 was still favorable for recovery of the correct structure, rather than collapsing into an alternative fold. Interestingly, while the average TM-score remained relatively unchanged after AlphaFold2 refinement for all diffusion steps, the average lDDT score consistently improved by around 0.04. This finding suggests that in this configuration, AlphaFold2 primarily refines local structures, with minimal adjustments to the protein backbone, as reflected by the stable TM-scores.

**Figure 5.**
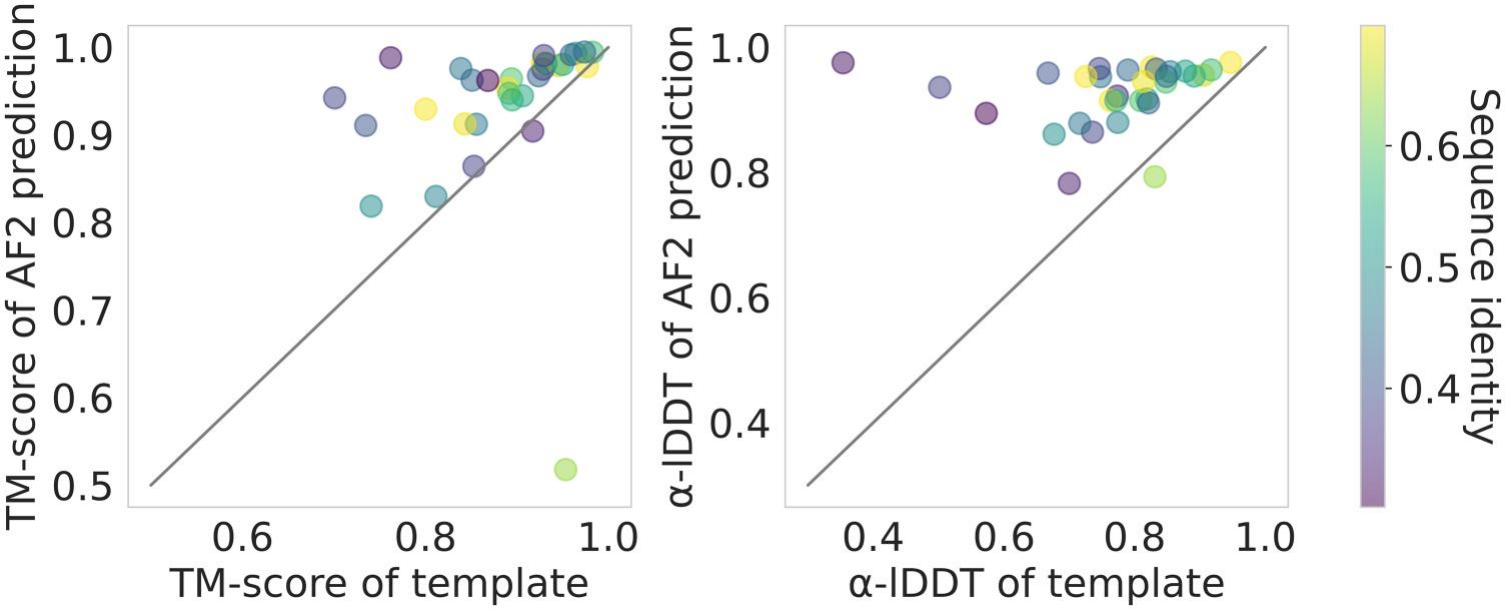
Impact of AlphaFold2 refinement in a homology modeling pipeline. Scatter plots illustrate the change in TM-score (left) and lDDT of Cα (right) after processing by AlphaFold2. Each point represents a single target from the CAMEO dataset, colored by sequence identity. The *x*-axis displays the scores of the templates compared to the target structures, and the *y*-axis shows the scores after refinement with AlphaFold2.

To evaluate AlphaFold2’s performance in a practical context, we integrated it into a homology modeling pipeline. For each protein structure in the CAMEO dataset, we searched for homologous proteins within the PDB70 structural library (Burley *et al.* 2017) and obtained suitable templates for 31 out of the 54 targets (Supporting Information Table A5). We observed that Alphafold2 was capable of consistently generating high-quality protein structure predictions, despite the template sequences not being exact matches (Fig. 5). The lowest-ranked prediction (PDB ID: 8DCE) corresponds to an artificial fusion protein composed of the fragments from the variable regions of the heavy and light chains of an immunoglobulin.

Previous research has shown that AlphaFold2 can estimate template quality more precisely by replacing all residues in the template with glycine extended with a Cβ, and providing the sequence with all gaps and an empty MSA (AF2Rank) (Roney and Ovchinnikov 2022). We implemented a similar method within OpenFold, which we term OF2Rank. Interestingly, while OF2Rank achieved lower performance compared to AlphaFold2 when recovering structures from templates perturbed with Gaussian noise or projected into 1D/2D PCA space, it showed comparable effectiveness for RFdiffusion-perturbed templates. Furthermore, the advantages of OF2Rank became more pronounced with increasing numbers of RFdiffusion steps. At 10 partial diffusion steps, OF2Rank increased the average lDDT reached by 0.03. Intriguingly, using an all-gap MSA yielded slightly better predictions compared to the single sequence MSA, mirroring observations from AF2Rank (Roney and Ovchinnikov 2022). The average difference between the all-gap and single sequence pipelines for both average TM-score and average lDDT was approximately 0.03 each. However, overall, AlphaFold2 pipeline generally outperformed OF2Rank for the refinement of perturbed templates.

Replacing the template input with prev_x had minimal impact on the predictions, with the TM-Score increasing from 0.38 to 0.43 (Supplementary Appendix F). Further tests with full MSAs and disabled prev_x showed no striking difference (TM-Score of 0.84 ± 0.15 for both with and without prev_x in CASP14).

## 4 Discussion

This study investigated capabilities and limitations of AlphaFold2’s ability to understand protein structures. We designed a series of experiments to investigate how AlphaFold2 handles both local features, like side-chain packing, and global features, like perturbations to the backbone.

Our findings suggest that Cβs are crucial for AlphaFold2 to recognize a template as a valid protein structure. When Cβs are present, AlphaFold2 prioritizes side-chain packing and only marginally alters the backbone if the phylogenic signal is weak. This can be leveraged as preprocessing pipeline that refines incomplete experimental structures by standardizing atom numbering and modeling missing residues. Additionally, it can be used to refine back-mapped coarse-grained models into atomistic models for molecular dynamics simulations (Wassenaar *et al.* 2014). Interestingly, a simple heuristic for Cβ placement achieves performance comparable to providing the original position of the Cβ. However, providing more side-chain information of seemingly lower quality, like the predefined conformations from the CHARMM36 force field, did not notably improve the side-chain packing performance. Furthermore, prepacking the template with dedicated side-chain packing algorithms like FASPR and AttnPacker only marginally impacted the final packing performance by AlphaFold2.

This would further suggest that AlphaFold2’s understanding of the protein structure relies heavily on the presence of stereochemically valid Cβ. Even when provided with detailed side-chain packing information from templates, the network appears to prioritize the Cβ distogram over the specific side-chain torsion angles from templates. This implies that the information needed to accurately pack side chains is effectively reconstructed from either the MSA or the distogram-based features. Consequently, when provided with a high-quality backbone structure, it appears that AlphaFold2 can effectively constrain and reason about side-chain conformations within the local reference frame assigned to each residue. A potential application of this insight is the standardization of experimental templates, which often contain inconsistencies such as missing or unlabeled atoms.

We also observed that AlphaFold2’s side-chain packing performance remained nearly consistent regardless of residue burial depth, while the performance dropped noticeably for surface residues when AlphaFold2 relied solely on the MSA. This finding underlines the importance of high-quality templates, especially when dealing with shallow MSAs.

Perturbing the input template with various methods revealed insightful details regarding AlphaFold2’s capabilities in recovering 3D protein structures. AlphaFold2 efficiently recovered structures perturbed with Gaussian noise, which primarily involves local adjustments to bond lengths and angles within residues. This ease of recovery hints at AlphaFold2 potentially having learnt a biophysical energy model for proper steric interactions, similar to classical optimization methods that utilize molecular force fields. Even more striking is AlphaFold2’s ability to recover details of a 3D protein structure from 2D-like templates. This suggests that AlphaFold2 can effectively navigate the transition from a limited structural representation to a full 3D structure. Research on OpenFold, a reimplementation of AlphaFold2, showed that it first predicts a 2D representation during early stages of training before transitioning to 3D (Ahdritz *et al.* 2024). This finding raises the intriguing possibility that AlphaFold2 has preserved a similar internal representation of a protein structure.

A striking difference in relative performance was observed between AlphaFold2 and the OF2Rank method when refining templates perturbed with Gaussian noise or projected into 1D/2D PCA space, compared to those perturbed with RFdiffusion. The key distinction lies in the nature of the perturbations. RFdiffusion typically introduces realistic modifications that maintain valid protein structures, whereas other methods often generate structures with steric clashes and other imperfections.

The markedly lower performance of OF2Rank on these unrealistic protein structures highlights a potential strength of the underlying approach. By struggling with these templates, OF2Rank might be more adept at identifying unreliable starting points. This aligns with the notion that AlphaFold2 was trained on the assumption of structurally sound templates. When presented with corrupted starting structures and lacking a reliable MSA for guidance, AlphaFold2’s performance will decline as it grapples with reconciling the conflicting information.

Our observations of minimal impact from structure recycling in AlphaFold2 align with the decision by AlphaFold3’s authors to remove this mechanism entirely (Abramson *et al.* 2024).

Prior research on AlphaFold2 using MSA-free protocols suggests the structure module might have learned a valid biophysical energy function, independent of MSAs (Roney and Ovchinnikov 2022). AlphaFold2 would then act as an unrolled optimizer and make iterative adjustments guided by the learned potential to find a low-energy state, corresponding to a refined protein structure. Our results support this hypothesis, but suggest limitations. This optimization likely operates within a restricted neighborhood that requires both the backbone and stereochemically valid Cβs. Additionally, the function appears most effective for local molecular interactions, as evidenced by its successful handling of side-chain packing and Gaussian noise perturbations.

Our work provides valuable guidance for users to critically evaluate AlphaFold2’s predictions and identify scenarios where complementary tools might be necessary. These insights pave the way for further exploration of existing methods or the development of novel strategies to overcome AlphaFold2’s limitations, ultimately leading to more robust and reliable protein structure prediction.

## Supplementary Material

vbae187_Supplementary_Data

## Data Availability

Data and implementation are available at https://github.com/ibmm-unibe-ch/template-analysis.
