# Supplementary material for: Dissecting AlphaFold2’s capabilities with limited sequence information
Source: Bioinform Adv. 2024 Nov 25;5(1):vbae187. doi: 10.1093/bioadv/vbae187 (PMC11751578; doi:10.1093/bioadv/vbae187)
Supplement: vbae187_Supplementary_Data [file vbae187_supplementary_data.pdf]

Table A1. Side-chain packing results

Method	Dataset	TM-score ↑	lDDT ↑	RMSD (Å) ↓	MAE 1 (rad) ↓	MAE 2 (rad) ↓	MAE 3 (rad) ↓	MAE 4 (rad) ↓
**MSA**	**CAMEO**	0.892 ± 0.166	0.831 ± 0.110	0.784 ± 0.276	0.479 ± 0.199	0.490 ± 0.145	0.805 ± 0.170	0.956 ± 0.223
	**CASP13**	0.831 ± 0.196	0.811 ± 0.113	0.799 ± 0.225	0.517 ± 0.188	0.500 ± 0.130	0.817 ± 0.163	0.947 ± 0.234
	**CASP14**	0.793 ± 0.178	0.743 ± 0.146	0.973 ± 0.254	0.632 ± 0.191	0.573 ± 0.149	0.882 ± 0.158	0.877 ± 0.229
**Template**	**CAMEO**	0.961 ± 0.132	0.934 ± 0.070	0.538 ± 0.282	0.284 ± 0.203	0.338 ± 0.162	0.605 ± 0.189	0.836 ± 0.179
	**CASP13**	0.985 ± 0.032	0.945 ± 0.038	0.499 ± 0.200	0.274 ± 0.150	0.316 ± 0.128	0.586 ± 0.181	0.797 ± 0.230
	**CASP14**	0.982 ± 0.031	0.926 ± 0.045	0.616 ± 0.235	0.350 ± 0.185	0.381 ± 0.162	0.649 ± 0.181	0.756 ± 0.235
**Backbone**	**CAMEO**	0.462 ± 0.227	0.430 ± 0.191	1.090 ± 0.201	0.708 ± 0.143	0.641 ± 0.101	0.895 ± 0.193	0.995 ± 0.173
	**CASP13**	0.411 ± 0.205	0.393 ± 0.168	1.107 ± 0.171	0.740 ± 0.140	0.630 ± 0.105	0.908 ± 0.143	0.959 ± 0.212
	**CASP14**	0.409 ± 0.200	0.392 ± 0.178	1.183 ± 0.198	0.784 ± 0.143	0.653 ± 0.107	0.932 ± 0.152	0.875 ± 0.234
**Non-informative Cβ**	**CAMEO**	0.346 ± 0.218	0.324 ± 0.198	1.156 ± 0.194	0.762 ± 0.138	0.671 ± 0.094	0.921 ± 0.187	0.994 ± 0.169
	**CASP13**	0.325 ± 0.200	0.306 ± 0.174	1.164 ± 0.168	0.786 ± 0.142	0.654 ± 0.103	0.915 ± 0.166	0.955 ± 0.222
	**CASP14**	0.318 ± 0.210	0.311 ± 0.189	1.238 ± 0.184	0.828 ± 0.137	0.673 ± 0.098	0.950 ± 0.163	0.876 ± 0.238
**Heuristic Cβ**	**CAMEO**	0.958 ± 0.132	0.898 ± 0.059	0.777 ± 0.258	0.460 ± 0.192	0.502 ± 0.152	0.837 ± 0.181	0.984 ± 0.166
	**CASP13**	0.979 ± 0.061	0.901 ± 0.038	0.793 ± 0.210	0.494 ± 0.177	0.506 ± 0.126	0.828 ± 0.164	0.960 ± 0.213
	**CASP14**	0.979 ± 0.034	0.883 ± 0.046	0.911 ± 0.252	0.575 ± 0.193	0.556 ± 0.151	0.846 ± 0.164	0.878 ± 0.242
**Template Cβ**	**CAMEO**	0.951 ± 0.134	0.894 ± 0.070	0.741 ± 0.272	0.441 ± 0.203	0.472 ± 0.133	0.805 ± 0.191	0.961 ± 0.174
	**CASP13**	0.974 ± 0.062	0.905 ± 0.041	0.738 ± 0.218	0.462 ± 0.176	0.465 ± 0.129	0.795 ± 0.170	0.936 ± 0.223
	**CASP14**	0.971 ± 0.038	0.883 ± 0.052	0.860 ± 0.265	0.544 ± 0.196	0.516 ± 0.164	0.842 ± 0.165	0.854 ± 0.241
**C36**	**CAMEO**	1.000 ± 0.000	0.783 ± 0.020	1.770 ± 0.192	1.357 ± 0.100	0.767 ± 0.122	1.006 ± 0.204	0.985 ± 0.180
	**CASP13**	1.000 ± 0.000	0.782 ± 0.017	1.731 ± 0.149	1.339 ± 0.086	0.732 ± 0.100	0.997 ± 0.187	0.951 ± 0.213
	**CASP14**	1.000 ± 0.000	0.786 ± 0.018	1.769 ± 0.154	1.315 ± 0.096	0.743 ± 0.121	0.990 ± 0.168	0.869 ± 0.240
**C36 (AF2)**	**CAMEO**	0.957 ± 0.131	0.898 ± 0.068	0.774 ± 0.262	0.461 ± 0.198	0.479 ± 0.132	0.830 ± 0.182	0.961 ± 0.174
	**CASP13**	0.962 ± 0.111	0.890 ± 0.099	0.778 ± 0.215	0.485 ± 0.177	0.475 ± 0.127	0.817 ± 0.191	0.948 ± 0.222
	**CASP14**	0.978 ± 0.036	0.888 ± 0.045	0.883 ± 0.249	0.552 ± 0.188	0.527 ± 0.153	0.849 ± 0.157	0.859 ± 0.242
**FASPR**	**CAMEO**	1.000 ± 0.000	0.923 ± 0.031	0.784 ± 0.255	0.457 ± 0.177	0.515 ± 0.120	0.854 ± 0.186	1.063 ± 0.294
	**CASP13**	1.000 ± 0.000	0.926 ± 0.027	0.756 ± 0.228	0.451 ± 0.167	0.488 ± 0.140	0.863 ± 0.180	1.017 ± 0.279
	**CASP14**	1.000 ± 0.000	0.911 ± 0.030	0.896 ± 0.265	0.548 ± 0.197	0.554 ± 0.168	0.860 ± 0.156	0.963 ± 0.246
**FASPR (AF2)**	**CAMEO**	0.963 ± 0.124	0.915 ± 0.061	0.717 ± 0.275	0.414 ± 0.198	0.468 ± 0.140	0.790 ± 0.197	0.972 ± 0.203
	**CASP13**	0.983 ± 0.049	0.924 ± 0.037	0.694 ± 0.231	0.418 ± 0.175	0.450 ± 0.140	0.801 ± 0.193	0.932 ± 0.238
	**CASP14**	0.983 ± 0.030	0.905 ± 0.043	0.833 ± 0.274	0.511 ± 0.206	0.514 ± 0.176	0.821 ± 0.153	0.888 ± 0.237
**AttnPacker**	**CAMEO**	1.000 ± 0.000	0.945 ± 0.030	0.600 ± 0.244	0.335 ± 0.174	0.431 ± 0.137	0.812 ± 0.159	0.987 ± 0.203
	**CASP13**	1.000 ± 0.000	0.953 ± 0.025	0.532 ± 0.202	0.302 ± 0.150	0.394 ± 0.127	0.773 ± 0.189	0.953 ± 0.226
	**CASP14**	1.000 ± 0.000	0.937 ± 0.026	0.687 ± 0.220	0.414 ± 0.176	0.480 ± 0.151	0.832 ± 0.159	0.891 ± 0.229
**AttnPacker (AF2)**	**CAMEO**	0.962 ± 0.128	0.921 ± 0.067	0.660 ± 0.285	0.364 ± 0.213	0.430 ± 0.154	0.756 ± 0.178	0.949 ± 0.178
	**CASP13**	0.986 ± 0.031	0.932 ± 0.039	0.616 ± 0.220	0.356 ± 0.175	0.406 ± 0.132	0.734 ± 0.186	0.921 ± 0.226
	**CASP14**	0.983 ± 0.030	0.910 ± 0.045	0.772 ± 0.263	0.461 ± 0.203	0.484 ± 0.163	0.790 ± 0.164	0.862 ± 0.235

Results of the side-chain packing experiment. Averages with standard deviation are shown for CAMEO, CASP13 and CASP14 separately. TM-score is used to score the backbone, while lDDT and RMSD score backbone and side-chains simultaneously and the mean absolute errors of dihedral side-chain angles in Radians, starting from the first angle to the fourth give exclusive side-chain results. AlphaFold2 refinement with: a full MSA and no template (*MSA*), full ground truth template (*Template*), just the backbone (*Backbone*), ground truth backbone and Cβ placed next to the origin *Non-informative Cβ*, ground truth backbone and the Cβ placed with a heuristic (*Heuristic Cβ*) and backbone and Cβ from ground truth template (*Template Cβ*), or ground truth backbone and side-chains placed with CHARMM 36 force field (*C36*), FASPR (*FASPR*) or AttnPacker (*AttnPacker*). The *(AF2)* suffix indicates AlphaFold2 post-processing after side-chain packing.

A Side-chain packing table

Table A1 shows the results of the different side-chain packing experiments. The scores are computed for each target independently, then average and standard deviation are determined over the target scores. The major results are discussed in Section 3.1 of the main text.

B Side-chain packing performance correlation with other evaluation measures

To further analyze the results of the side-chain packing experiment, a comparison with confidence (pLDDT) and their relative accessible surface area (rASA)(Shrake and Rupley, 1973; Touw et al., 2015) is performed. The residues are binned into 11 bins depending on the rASA rounded to the first decimal in the ground truth target. Therefore, the histogram for CASP13, depicted in Figure A1, and CASP14, shown in Figure A2, is the same for each side-chain packing method with the same dataset. A plot comparing rASA with pLDDT and lDDT

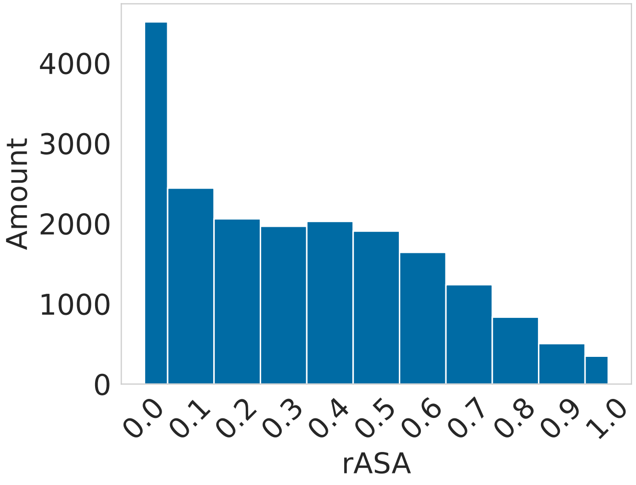

Fig. A1. Histogram of rASA bins on the CASP13 dataset.

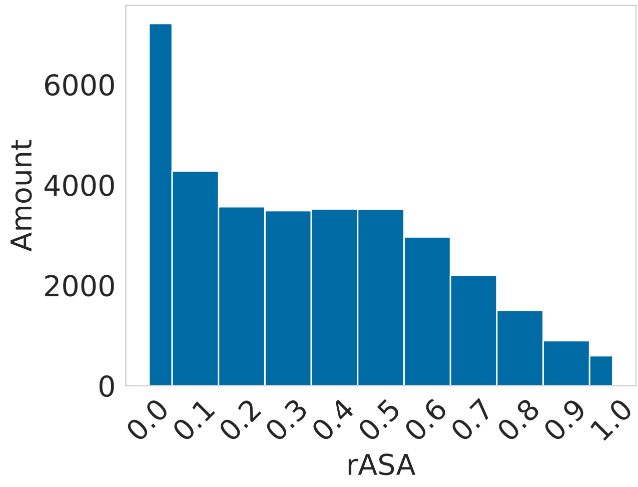

Fig. A2. Histogram of rASA bins on the CASP14 dataset.

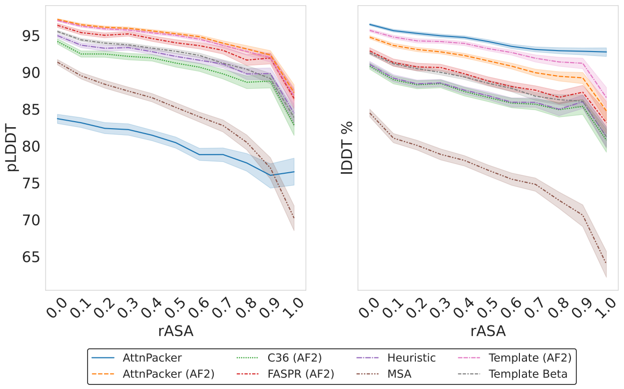

Fig. A3. Relationship between rASA and pLDDT and lDDT for the CASP13 dataset. The line indicates the average score and the shadow area shows the 95% confidence interval. Side-chain packing using: ground truth backbone and side-chains placed with AttnPacker (*AttnPacker*), CHARMM 36 force field (*C36*) or FASPR (*FASPR*), ground truth backbone and the Cβ placed with a heuristic (*Heuristic*), AlphaFold2 with a full MSA and no template (*MSA*), full ground truth template (*Template (AF2)*), backbone and Cβ from ground truth template (*Template Beta*). The *(AF2)* suffix indicates AlphaFold2 post-processing after side-chain packing.

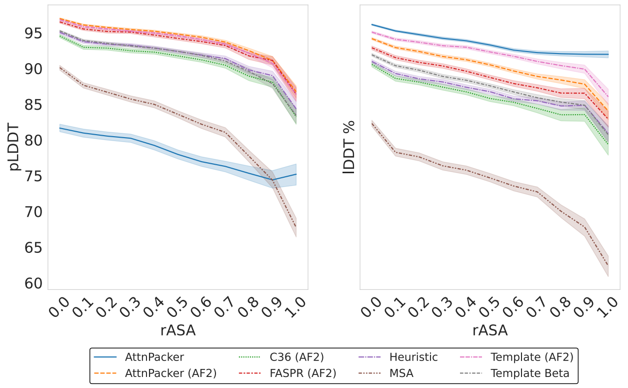

Fig. A4. Relationship between rASA and pLDDT and lDDT for the CASP14 dataset.The line indicates the average score and the shadow area shows the 95% confidence interval. Side-chain packing using: ground truth backbone and side-chains placed with AttnPacker (*AttnPacker*), CHARMM 36 force field (*C36*) or FASPR (*FASPR*), ground truth backbone and the Cβ placed with a heuristic (*Heuristic*), AlphaFold2 with a full MSA and no template (*MSA*), full ground truth template (*Template (AF2)*), backbone and Cβ from ground truth template (*Template Beta*). The *(AF2)* suffix indicates AlphaFold2 post-processing after side-chain packing.

Table A2. Pearson correlations of lDDT, pLDDT and rASA

Method	Dataset	lDDT×rASA	pLDDT×rASA	lDDT×pLDDT
**MSA**	**CASP13**	-0.276	-0.313	0.786
	**CASP14**	-0.256	-0.301	0.784
**Template**	**CASP13**	-0.247	-0.310	0.697
	**CASP14**	-0.257	-0.300	0.686
**Heuristic Cβ**	**CASP13**	-0.198	-0.211	0.779
	**CASP14**	-0.230	-0.230	0.728
**Template Cβ**	**CASP13**	-0.302	-0.318	0.656
	**CASP14**	-0.300	-0.303	0.649
**C36 (AF2)**	**CASP13**	-0.182	-0.179	0.681
	**CASP14**	-0.204	-0.203	0.633
**FASPR (AF2)**	**CASP13**	-0.209	-0.199	0.811
	**CASP14**	-0.242	-0.225	0.758
**AttnPacker**	**CASP13**	-0.265	-0.155	0.341
	**CASP14**	-0.291	-0.154	0.388
**AttnPacker (AF2)**	**CASP13**	-0.285	-0.311	0.644
	**CASP14**	-0.298	-0.301	0.637

Pearson correlations between lDDT, pLDDT and rASA for CASP13 and CASP14. AlphaFold2 refinement with: a full MSA and no template (*MSA*), full ground truth template (*Template*), ground truth backbone and the Cβ placed with a heuristic (*Heuristic Cβ*), backbone and Cβ from ground truth template (*Template Cβ*) or ground truth backbone and side-chains placed with CHARMM 36 force field (*C36 (AF2)*), FASPR or (*FASPR (AF2)*). Additionally, AttnPacker was evaluated before (*AttnPacker*) and after (*AttnPacker (AF2)*) AlphaFold2 refinement.

on the CASP13 dataset can be seen in Figure A3 and a similar plot for CASP14 can be found in Figure A4.

As expected, the pLDDT and lDDT drops with increasing rASA on average. This drop is only minor until the bins at around a rASA of 0.9, where the drop becomes steeper. Comparing the different packers, the majority of them stay pretty well together; their scores are ordered the same as in the results in Section 3.1. The lDDT and pLDDT have very similar curves, which indicates that pLDDT is also a good lDDT estimator in these circumstances. The exact Pearson correlation coefficients are reported in Table A2 between lDDT, pLDDT and rASA on the CASP13 and the CASP14 dataset for multiple side-chain placement protocols.

The first outlier to the majority is AttnPacker, which generally has a lower confidence, but a higher lDDT. The reason for the lower score in the pLDDT figure is due that AttnPacker reports its own confidence, which is not the same as the confidence from AlphaFold2. The higher lDDT score then can be explained by a superior packing performance and that the backbone, which influences this metric, did not get modified.

The other outlier is standard AlphaFold2 using the full multiple sequence alignment (MSA). This setup performs worse than the backbone informed methods, but the pLDDT and lDDT still have a good correlation.

C Refinement tables

The major results of the refinement task are discussed in Section 3.2 in the main text. Table A3 shows results for Gaussian noise and principal component reduction, while Table A4 displays the results of RFdiffusion. The scores are computed

Table A3. Refinement results on Gaussian noise and principal components perturbation

Method	Dataset	TM-score ↑	lDDT ↑	α-RMSD (Å) ↓
**MSA**	**CAMEO**	0.895±0.156	0.843±0.113	3.217±6.578
	**CASP13**	0.858±0.162	0.833±0.095	4.627±5.258
	**CASP14**	0.840±0.147	0.791±0.116	4.841±5.207
**Single**	**CAMEO**	0.415±0.201	0.362±0.182	17.863±10.866
	**CASP13**	0.370±0.144	0.335±0.127	20.434±11.086
	**CASP14**	0.366±0.168	0.336±0.152	19.620±9.652
**Gaussian**	**CAMEO**	0.882±0.118	0.659±0.005	1.703±0.069
	**CASP13**	0.900±0.053	0.659±0.003	1.710±0.055
	**CASP14**	0.896±0.052	0.659±0.004	1.712±0.058
**Gaussian (AF2)**	**CAMEO**	0.927±0.121	0.839±0.069	1.755±1.717
	**CASP13**	0.935±0.075	0.839±0.054	2.235±4.849
	**CASP14**	0.922±0.062	0.817±0.070	1.994±1.454
**Gaussian (OF2Rank Single)**	**CAMEO**	0.845±0.135	0.712±0.093	3.434±2.160
	**CASP13**	0.682±0.233	0.611±0.185	10.457±11.728
	**CASP14**	0.686±0.207	0.594±0.179	9.344±8.205
**Gaussian (OF2Rank Empty)**	**CAMEO**	0.849±0.145	0.723±0.097	3.204±2.246
	**CASP13**	0.708±0.234	0.627±0.194	10.159±12.098
	**CASP14**	0.717±0.214	0.621±0.186	8.879±9.597
**1 PC**	**CAMEO**	0.212±0.043	0.243±0.034	12.622±3.533
	**CASP13**	0.215±0.036	0.238±0.035	14.756±8.037
	**CASP14**	0.228±0.053	0.245±0.036	13.787±5.669
**1 PC (AF2)**	**CAMEO**	0.519±0.251	0.441±0.228	15.357±13.016
	**CASP13**	0.458±0.202	0.392±0.184	18.466±13.492
	**CASP14**	0.443±0.213	0.394±0.204	18.000±12.469
**1 PC (OF2Rank Single)**	**CAMEO**	0.461±0.193	0.393±0.187	15.476±8.861
	**CASP13**	0.402±0.150	0.358±0.134	19.131±10.953
	**CASP14**	0.412±0.170	0.368±0.163	18.504±10.069
**1 PC (OF2Rank Empty)**	**CAMEO**	0.460±0.192	0.391±0.186	15.016±8.330
	**CASP13**	0.402±0.160	0.352±0.141	19.566±11.392
	**CASP14**	0.398±0.172	0.357±0.156	18.756±10.241
**2 PC**	**CAMEO**	0.483±0.059	0.562±0.061	8.308±2.809
	**CASP13**	0.464±0.077	0.535±0.084	9.884±6.939
	**CASP14**	0.487±0.075	0.534±0.090	8.786±3.749
**2 PC (AF2)**	**CAMEO**	0.928±0.119	0.851±0.067	1.804±1.703
	**CASP13**	0.838±0.212	0.792±0.166	5.715±11.525
	**CASP14**	0.862±0.149	0.793±0.124	3.779±5.227
**2 PC (OF2Rank Single)**	**CAMEO**	0.742±0.168	0.616±0.132	6.179±4.212
	**CASP13**	0.596±0.227	0.531±0.183	13.423±12.195
	**CASP14**	0.575±0.201	0.499±0.179	11.568±7.817
**2 PC (OF2Rank Empty)**	**CAMEO**	0.804±0.137	0.672±0.110	4.446±2.975
	**CASP13**	0.626±0.235	0.558±0.190	12.417±12.380
	**CASP14**	0.618±0.211	0.536±0.185	10.445±7.978

Results for the perturbation experiment with Gaussian noise and principal components perturbation. Averages with standard deviation are shown for CAMEO, CASP13 and CASP14 separately. TM-score and Cα RMSD in Å are used to score the backbone, while lDDT scores backbone and side-chains simultaneously. AlphaFold2 run with: a full MSA and no template (*MSA*), single sequence and no template (*Single*). The *(AF2)* suffix is used to indicate AlphaFold2 post-processing. *(OF2Rank Single)* and *(OF2Rank Empty)* note the use of the AF2Rank inspired pipeline with a single sequence or an all gap MSA respectively. *Gaussian* perturbes the template with Gaussian noise, *1 PC* reduces the template to the first principal component, *2 PC* reduces the template to the first two principal components.

for each target independently, then average and standard deviation are determined over the target scores.

D Homology modeling targets

MMseqs2 (Mirdita et al., 2021) was used to select one template per CAMEO (Robin et al., 2021) protein from the PDB databank (Burley et al., 2017) with a sequence identity between 30% and 70% and a sequence coverage of at least 80%. This process yielded suitable templates for 31 of the 54 targets listed in Table A5.

E Error analysis

To gain a better understanding of the behaviour of the template based predictions, we compared the folding difficulty by using pLDDT of the standard MSA pipeline from AlphaFold2 and accuracy of the prediction using the correct template through lDDT. Additionally, the secondary structures were annotated with DSSP(Touw et al., 2015). A subset of 200 residues from the CASP14 dataset were randomly picked.

It can be observed in Figure A5 that given the correct template, AlphaFold2 performs good to very good at protein folding for the vast majority of residues; independent of difficulty of protein or secondary structure.

Table A4. Refinement results on RFdiffusion perturbation

Method	Dataset	TM-score ↑	lDDT ↑	α-RMSD (Å) ↓
**MSA**	**CAMEO**	0.895±0.156	0.843±0.113	3.217±6.578
	**CASP13**	0.858±0.162	0.833±0.095	4.627±5.258
	**CASP14**	0.840±0.147	0.791±0.116	4.841±5.207
**Single**	**CAMEO**	0.415±0.201	0.362±0.182	17.863±10.866
	**CASP13**	0.370±0.144	0.335±0.127	20.434±11.086
	**CASP14**	0.366±0.168	0.336±0.152	19.620±9.652
**1 RFDiff (FASPR)**	**CAMEO**	0.961±0.102	0.819±0.025	0.847±0.620
	**CASP13**	0.970±0.037	0.810±0.028	0.955±0.804
	**CASP14**	0.974±0.013	0.804±0.026	0.851±0.268
**1 RFDiff (FASPR AF2)**	**CAMEO**	0.948±0.134	0.856±0.053	1.249±2.416
	**CASP13**	0.959±0.074	0.844±0.085	1.418±2.510
	**CASP14**	0.968±0.027	0.842±0.043	1.007±0.575
**1 RFDiff (AP)**	**CAMEO**	0.961±0.102	0.841±0.026	0.847±0.620
	**CASP13**	0.970±0.037	0.836±0.028	0.955±0.804
	**CASP14**	0.974±0.013	0.827±0.028	0.851±0.268
**1 RFDiff (AP AF2)**	**CAMEO**	0.948±0.134	0.863±0.060	1.270±2.479
	**CASP13**	0.961±0.072	0.855±0.087	1.414±2.548
	**CASP14**	0.967±0.028	0.853±0.044	1.026±0.543
**1 RFDiff (OF2Rank Single)**	**CAMEO**	0.888±0.139	0.786±0.069	2.721±2.664
	**CASP13**	0.845±0.162	0.773±0.089	5.754±10.510
	**CASP14**	0.853±0.107	0.754±0.087	4.066±4.322
**1 RFDiff (OF2Rank Empty)**	**CAMEO**	0.902±0.139	0.812±0.069	2.413±2.670
	**CASP13**	0.883±0.128	0.809±0.063	4.668±9.710
	**CASP14**	0.881±0.095	0.785±0.084	3.213±3.579
**5 RFDiff (FASPR)**	**CAMEO**	0.922±0.112	0.737±0.036	1.502±0.821
	**CASP13**	0.918±0.063	0.725±0.037	1.826±1.684
	**CASP14**	0.927±0.032	0.723±0.035	1.524±0.452
**5 RFDiff (FASPR AF2)**	**CAMEO**	0.929±0.123	0.789±0.052	1.443±1.119
	**CASP13**	0.919±0.089	0.768±0.083	2.561±5.798
	**CASP14**	0.933±0.036	0.772±0.049	1.536±0.682
**5 RFDiff (AP)**	**CAMEO**	0.922±0.112	0.754±0.037	1.502±0.821
	**CASP13**	0.918±0.063	0.745±0.036	1.826±1.684
	**CASP14**	0.927±0.032	0.742±0.037	1.524±0.452
**5 RFDiff (AP AF2)**	**CAMEO**	0.931±0.120	0.801±0.051	1.413±1.012
	**CASP13**	0.922±0.085	0.781±0.084	2.059±2.511
	**CASP14**	0.935±0.036	0.785±0.048	1.529±0.665
**5 RFDiff (OF2Rank Single)**	**CAMEO**	0.883±0.135	0.777±0.071	2.802±2.281
	**CASP13**	0.832±0.164	0.749±0.095	6.035±10.648
	**CASP14**	0.842±0.102	0.743±0.082	4.080±4.178
**5 RFDiff (OF2Rank Empty)**	**CAMEO**	0.903±0.126	0.801±0.063	2.284±1.784
	**CASP13**	0.873±0.125	0.788±0.066	5.211±11.156
	**CASP14**	0.868±0.091	0.767±0.078	3.192±2.870
**10 RFDiff (FASPR)**	**CAMEO**	0.882±0.120	0.686±0.045	2.070±0.876
	**CASP13**	0.867±0.072	0.669±0.047	2.501±1.683
	**CASP14**	0.873±0.052	0.670±0.046	2.191±0.554
**10 RFDiff (FASPR AF2)**	**CAMEO**	0.898±0.120	0.740±0.068	1.980±1.323
	**CASP13**	0.874±0.092	0.709±0.088	2.806±2.978
	**CASP14**	0.884±0.053	0.715±0.059	2.166±0.689
**10 RFDiff (AP)**	**CAMEO**	0.882±0.120	0.701±0.048	2.070±0.876
	**CASP13**	0.867±0.072	0.685±0.048	2.501±1.683
	**CASP14**	0.873±0.052	0.684±0.048	2.191±0.554
**10 RFDiff (AP AF2)**	**CAMEO**	0.902±0.121	0.752±0.065	1.915±1.290
	**CASP13**	0.879±0.092	0.721±0.089	2.667±2.473
	**CASP14**	0.889±0.050	0.728±0.059	2.109±0.658
**10 RFDiff (OF2Rank Single)**	**CAMEO**	0.878±0.125	0.763±0.078	2.869±1.916
	**CASP13**	0.818±0.158	0.733±0.094	6.250±11.003
	**CASP14**	0.817±0.109	0.712±0.095	4.801±4.616
**10 RFDiff (OF2Rank Empty)**	**CAMEO**	0.892±0.124	0.781±0.072	2.526±1.863
	**CASP13**	0.854±0.128	0.764±0.077	5.421±10.890
	**CASP14**	0.844±0.099	0.742±0.089	3.597±2.951

Results for the perturbation experiment with partial RFdiffusion. Averages with standard deviation are shown for CAMEO, CASP13 and CASP14 separately. TM-score and RMSD of the Cα in Å are used to score the backbone, while lDDT scores backbone and side-chains simultaneously. AlphaFold2 baselines ran with: a full MSA and no template *MSA*, or single sequence and no template *Single*. The *(AF2)* suffix is used to indicate AlphaFold2 post-processing. *(OF2Rank Single)* and *(OF2Rank Empty)* note the use of the AF2Rank inspired pipeline with a single sequence or an all gap MSA respectively. *N RFDiff* perturbs the template by doing *N* partial diffusion steps. To pack side-chains to the diffused backbone, either FASPR (*FASPR*) or AttnPacker (*AP*) were used.

Table A5. Templates for homology modeling found with MMSeqs

Query	Hit	Seq. id.(%)	Query cov.(%)
8QJ5_A	3VSR_A	38.1	95.6
8QLU_A	7NJH_A	69.8	100.0
8RIU_C	1YTL_A	45.6	98.3
8RIU_D	3CF4_A	57.8	99.3
8ROH_A	6ZZ6_B	47.8	98.1
8S4H_A	4F9L_D	69.9	100.0
8VK9_D	6D68_C	69.9	100.0
8VZI_D	6XN8_A	42.4	99.1
8W14_A	2FSX_A	40.3	81.6
8W9U_A	3L8U_A	53.5	96.7
8WC0_B	6LHX_A	49.7	98.1
8WCF_A	6LHY_B	38.1	83.1
8WCG_C	6Z1Z_A	69.9	100.0
8WDM_B	2OGT_A	58.7	98.6
8WDQ_A	6TQO_T	55.7	97.4
8WE5_A	5YH4_A	48.4	98.4
8WEO_C	5OCL_A	69.1	95.2
8WEU_A	3R44_A	37.5	99.6
8WFH_E	8DCE_H	63.7	100.0
8WKC_A	7V8O_A	52.7	98.7
8WL1_A	2EW2_A	43.6	99.0
8WZC_A	3V32_B	58.5	97.5
8XYV_A	5CWM_A	31.9	85.9
8YJO_A	1R8W_A	44.2	99.7
8Z4Q_A	7Y3W_A	30.2	92.2
8ZJ5_A	4ATY_A	45.5	96.5
9AUE_A	6CBK_A	34.5	95.7
9CEL_D	5D3Z_A	42.6	98.5
9DDY_A	4LFE_A	54.1	97.5
9J91_A	6EE5_A	33.1	96.9
9J9A_A	5C2Z_A	67.0	97.2

Query CAMEO structure and corresponding hits from the PDB with highest sequence identity (*Seq. id.*) below 70% and a query coverage (*Query cov.*) of at least 80%.

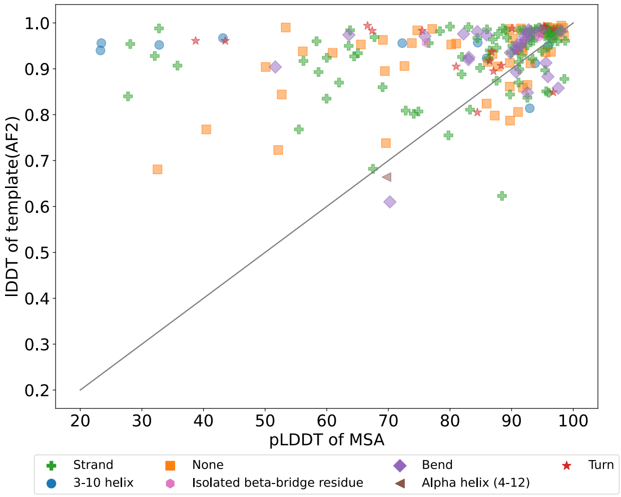

Fig. A5. Relationship between pLDDT score of predicted structures using full MSA and lDDT the single sequence and the correct template based predictions. The secondary structure defined by DSSP (Touw et al., 2015) of 200 uniformly sampled residues from CASP14 are shown with different symbols.

Table A6. Refinement results with prev_x modifications

Method	Dataset	TM-score ↑	lDDT ↑	α-RMSD (Å) ↓
**MSA**	**CASP13**	0.848±0.172	0.835±0.105	4.729±5.573
	**CASP14**	0.843±0.149	0.797±0.116	4.859±5.627
**Single**	**CASP13**	0.379±0.153	0.347±0.137	20.169±12.025
	**CASP14**	0.386±0.183	0.356±0.167	18.898±10.186
**OF prev_x**	**CASP13**	0.428±0.164	0.379±0.145	18.949±12.108
	**CASP14**	0.430±0.197	0.385±0.187	16.953±9.927
**OF no prev_x**	**CASP13**	0.853±0.180	0.832±0.113	4.914±6.223
	**CASP14**	0.848±0.146	0.794±0.116	4.732±5.885

Results for the prev_X experiments. Averages with standard deviation are shown for CASP13 and CASP14 separately. TM-score and RMSD of the Cα in Å are used to score the backbone, while lDDT scores backbone and side-chains simultaneously. AlphaFold2 baselines ran with: a full MSA and no template *MSA*, or single sequence and no template *Single*. *OF prev_x* indicates the results of an modified OpenFold version, where the ground truth template has been given as input for recycle 0 and the single sequence. An OpenFold version, where the prev_x input is completely disabled and the standard amount of three iterations is run on a full MSA is shown in *OF no prev_x*.

F Prev_x experiments

Since prev_x requires complete structures, all PDB entries with missing residues were excluded from the analysis presented in Table A6. This filtering resulted in a dataset of 60 structures for CASP13 and 48 structures for CASP14. For comparability, the scores for the other experiments were recomputed for this subset.

These experiments were conducted using a custom build of OpenFold. This version utilizes pre-trained weights and offers the ability to disable the prev_x output or provide a structure for the recycling input during the first pass. Additionally, it uses default embeddings for the MSA and pairwise representations.
